# Supplementary material for: Reasons for encounter in primary care among French patients: Gender differences in presentation rates more pronounced among the patients of male practitioners
Source: PLoS One. 2026 Apr 15;21(4):e0345781. doi: 10.1371/journal.pone.0345781 (PMC13082612; doi:10.1371/journal.pone.0345781)
Supplement: S1 Table — Pairwise analyses. (DOCX) [file pone.0345781.s001.docx]

**S1 Table.** Distribution of Reasons for encounter by GP/patient gender dyad. Pairwise analyses.

| Reasons for encounter  (Symptoms/complaints) | Gender dyad | n (%) | OR ^a^ [95] | P |
| --- | --- | --- | --- | --- |
| General and Unspecified | M/M*  M/F  F/M  F/F | 735 (12.0)  1014 (13.6)  374 (14.9)  638 (14.0) | 1  1.22 [1.09 - 1.35]  1.22 [0.99 - 1.49]  1.25 [1.04 - 1.52] | **0.002** |
| Digestive | M/M*  M/F  F/M  F/F | 679 (11.1)  916 (12.3)  313 (12.5)  610 (13.4) | 1  1.11 [1.00 - 1.24]  1.08 [0.90 - 1.29]  1.19 [1.01 - 1.40] | 0.09 |
| Eye | M/M*  M/F  F/M  F/F | 83 (1.4)  136 (1.8)  44 (1.8)  79 (1.7) | 1  1.40 [1.06 - 1.85]  1.24 [0.83 - 1.84]  1.32 [0.93 - 1.87] | 0.12 |
| Ear | M/M*  M/F  F/M  F/F | 151 (2.5)  216 (2.9)  68 (2.7)  146 (3.2) | 1  1.28 [1.04 - 1.59]  1.07 [0.78 - 1.50]  1.47 [1.10 - 1.96] | **0.01** |
| Circulatory | M/M*  M/F  F/M  F/F | 94 (1.5)  146 (2.0)  34 (1.4)  54 (1.2) | 1  1.23 [0.94 - 1.61]  0.97 [0.60 - 1.55]  0.78 [0.51 - 1.19] | 0.11 |
| Musculoskeletal | M/M*  M/F  F/M  F/F | 1038 (17.0)  1336 (17.9)  416 (16.6)  843 (18.5) | 1  1.02 [0.93 - 1.12]  0.92 [0.77 - 1.10]  0.94 [0.80 - 1.11] | 0.69 |
| Neurological | M/M*  M/F  F/M  F/F | 288 (4.7)  469 (6.3)  135 (5.4)  275 (6.1) | 1  1.31 [1.13 - 1.53]  1.05 [0.79 - 1.39]  1.05 [0.81 - 1.37] | **0.005** |
| Psychological | M/M*  M/F  F/M  F/F | 313 (5.1)  427 (5.7)  125 (5.0)  283 (6.2) | 1  1.08 [0.92 - 1.26]  0.92 [0.68 - 1.24]  0.99 [0.75 - 1.30] | 0.64 |
| Respiratory | M/M*  M/F  F/M  F/F | 1235 (20.2)  1549 (20.8)  559 (22.2)  944 (20.8) | 1  1.12 [1.03 - 1.22]  1.12 [0.94 - 1.33]  1.14 [1.97 - 1.34] | 0.06 |
