# Supplementary material for: Reasons for encounter in primary care among French patients: Gender differences in presentation rates more pronounced among the patients of male practitioners
Source: PLoS One. 2026 Apr 15;21(4):e0345781. doi: 10.1371/journal.pone.0345781 (PMC13082612; doi:10.1371/journal.pone.0345781)
Supplement: Attachment — Submitted filename: Response to Reviewers.pdf [file pone.0345781.s003.pdf]

Journal Requirements:

When submitting your revision, we need you to address these additional requirements.
1. Please ensure that your manuscript meets PLOS ONE's style requirements, including those for file naming. The PLOS ONE style templates can be found at https://journals.plos.org/plosone/s/file?id=wjVg/PLOSOne_formatting_sample_main_body.pdf and https://journals.plos.org/plosone/s/file?id=ba62/PLOSOne_formatting_sample_title_authors_affiliations.pdf

2. Please provide additional details regarding participant consent. In the ethics statement in the Methods and online submission information, please ensure that you have specified (1) whether consent was informed and (2) what type you obtained (for instance, written or verbal, and if verbal, how it was documented and witnessed). If your study included minors, state whether you obtained consent from parents or guardians. If the need for consent was waived by the ethics committee, please include this information.

Thank you for these important remarks. We have added information about patients' consent to participate in the study:

[Line 113 from the Methods section/Study design] :
Patients were given oral and visual information about the study, including the presence of the intern. Verbal consent was obtained from patients or the parents of minor participants. Patients could refuse the intern's presence while still participating in the study, or refuse both. Visits for which patients refused participation were excluded.
[Line 122 from the Methods section/Study design]
A declaration was filed with the former Advisory Committee on the Processing of Information in Health Research (CCTIRS, active at the time ECOGEN was designed) and then with the National Commission for Information Technology and Civil Liberties (CNIL) – both advisory boards approved the research methodology. Further, the ECOGEN survey was approved by Ethics Committee Sud-Est IV (No.L11-149), which explicitly approved the oral form of consent obtained from patients

3. In this instance it seems there may be acceptable restrictions in place that prevent the public sharing of your minimal data. However, in line with our goal of ensuring long-term data availability to all interested researchers, PLOS' Data Policy states that authors cannot be the sole named individuals responsible for ensuring data access (http://journals.plos.org/plosone/s/data-availability#loc-acceptable-data-sharing-methods).

Data requests to a non-author institutional point of contact, such as a data access or ethics committee, helps guarantee long term stability and availability of data. Providing interested researchers with a durable point of contact ensures data will be accessible even if an author changes email addresses, institutions, or becomes unavailable to answer requests.

Before we proceed with your manuscript, please also provide non-author contact information (phone/email/hyperlink) for a data access committee, ethics committee, or other institutional

body to which data requests may be sent. If no institutional body is available to respond to requests for your minimal data, please consider if there any institutional representatives who did not collaborate in the study, and are not listed as authors on the manuscript, who would be able to hold the data and respond to external requests for data access? If so, please provide their contact information (i.e., email address). Please also provide details on how you will ensure persistent or long-term data storage and availability.

Thank you for this remark. The data is available by request at the Université Claude Bernard Lyon 1 (UCBL), where they are stored on the UCBL servers.

4. We note that the grant information you provided in the 'Funding Information' and 'Financial Disclosure' sections do not match.
When you resubmit, please ensure that you provide the correct grant numbers for the awards you received for your study in the 'Funding Information' section.

Thank you for this remark. We have modified our statements and added the information to the article.

[Ligne 359 from Disclosure statement]
"The ECOGEN study was supported by the French National College of teachers in general practice via a grant from Pfizer laboratories (grant number not applicable), received by Laurent Letrillart. Eugenia Alcalde was funded by the Gender and Health Inequalities (GENDHI) project (grant agreement No. [ERC-2019-SyG n° 856478]). This funding had no role in study design, data collection and analysis, decision to publish, or preparation of the manuscript."

5. Thank you for stating the following financial disclosure:
"The author(s) received no specific funding for this work."
At this time, please address the following queries:
a) Please clarify the sources of funding (financial or material support) for your study. List the grants or organizations that supported your study, including funding received from your institution.
b) State what role the funders took in the study. If the funders had no role in your study, please state: "The funders had no role in study design, data collection and analysis, decision to publish, or preparation of the manuscript."
c) If any authors received a salary from any of your funders, please state which authors and which funders.
d) If you did not receive any funding for this study, please state: "The authors received no specific funding for this work."
Please include your amended statements within your cover letter; we will change the online submission form on your behalf.

Thank you for this remark.

a) The ECOGEN study was supported by the French National College of teachers in general practice via a grant from Pfizer laboratories (grant number not applicable), received by Laurent Letrillart.

Eugenia Alcalde was funded by the Gender and Health Inequalities (GENDHI) project (grant agreement No. [ERC-2019-SyG n° 856478]). This funding had no role in study design, data collection and analysis, decision to publish, or preparation of the manuscript.
The rest of the authors had no relevant or material financial interests that relate to the research described in this paper

b) The funders had no role in study design, data collection and analysis, decision to publish, or preparation of the manuscript.
c) None of the authors received a salary from the funders.

We have included the amended statement to our revised cover letter.

6. If the reviewer comments include a recommendation to cite specific previously published works, please review and evaluate these publications to determine whether they are relevant and should be cited. There is no requirement to cite these works unless the editor has indicated otherwise.

7. Please review your reference list to ensure that it is complete and correct. If you have cited papers that have been retracted, please include the rationale for doing so in the manuscript text, or remove these references and replace them with relevant current references. Any changes to the reference list should be mentioned in the rebuttal letter that accompanies your revised manuscript. If you need to cite a retracted article, indicate the article's retracted status in the References list and also include a citation and full reference for the retraction notice.

[Note: HTML markup is below. Please do not edit.]

Reviewers' comments:

Reviewer's Responses to Questions
Comments to the Author

1. Is the manuscript technically sound, and do the data support the conclusions?

The manuscript must describe a technically sound piece of scientific research with data that supports the conclusions. Experiments must have been conducted rigorously, with appropriate controls, replication, and sample sizes. The conclusions must be drawn appropriately based on the data presented.
Reviewer #1: Partly
Reviewer #2: Partly
Reviewer #3: Yes

---

2. Has the statistical analysis been performed appropriately and rigorously?
Reviewer #1: Yes
Reviewer #2: Yes
Reviewer #3: Yes

3. Have the authors made all data underlying the findings in their manuscript fully available?

The PLOS Data policy requires authors to make all data underlying the findings described in their manuscript fully available without restriction, with rare exception (please refer to the Data Availability Statement in the manuscript PDF file). The data should be provided as part of the manuscript or its supporting information, or deposited to a public repository. For example, in addition to summary statistics, the data points behind means, medians and variance measures should be available. If there are restrictions on publicly sharing data—e.g. participant privacy or use of data from a third party—those must be specified.
Reviewer #1: Yes
Reviewer #2: Yes
Reviewer #3: Yes

---

4. Is the manuscript presented in an intelligible fashion and written in standard English?

PLOS ONE does not copyedit accepted manuscripts, so the language in submitted articles must be clear, correct, and unambiguous. Any typographical or grammatical errors should be corrected at revision, so please note any specific errors here.
Reviewer #1: Yes
Reviewer #2: Yes
Reviewer #3: Yes

---

5. Review Comments to the Author

Please use the space provided to explain your answers to the questions above. You may also include additional comments for the author, including concerns about dual publication, research ethics, or publication ethics. (Please upload your review as an attachment if it exceeds 20,000 characters)

Reviewer #1: The study topic is good, but it is not completely clear and needs to be clarified, especially with regard to the study summary.
The study needs to detail the methodology, the summary, and even the results, and why gender affects medical issues.

We'd like to thank the reviewer for this global comment. We agree that our summary lacks clarity. We have modified all sections as follows:

Background: Evidence has shown that the gender of general practitioners (GPs) and of patients plays a role in primary care, both in communication and practice style. However, less is known about how both genders interact in the reasons for encounter (RfE). Studying RfEs by both patient and GP gender may help elucidate what symptoms or complaints lead women and men to consult a GP and specifically, if patients have preferences regarding the gender of their GP for specific problems. Given that men tend to consult less frequently and are often diagnosed at later stages of disease, it is essential to disentangle whether GP gender may act as a barrier for men in disclosing certain health concerns.

Then, in the methods section:

Methods: This is an ancillary study from the French ECOGEN survey, an observational cross-sectional multicentre study conducted between November 2011 and April 2012. 54 GP interns working alongside 128 GP supervisors collected RfEs during consultations. Using mixed models, we first assessed the association between patient gender and each RfE chapter (i.e., 13 independent variables) among all patients. We then stratified models according to GP gender. Finally, we compared the four physician–patient gender dyads (male physician–male patient, male physician–female patient, female physician–male patient, and female physician–female patient) in pairwise analyses.

The results section:

Results: Overall, 20 613 patients were included, of whom 58% were women. Of the 128 participating GPs, 85 were men. Women patients had higher presentation rates across all chapters. The most common RfE were those related to the respiratory system. Modest differences in presentation rates were observed between men and women when visiting male GPs, with men being less likely to present RfEs from the General and Unspecified, Digestive, Eyes, Ears, Neurological, and Respiratory chapters.

And finally the conclusion statement:

Conclusions: Our study adds evidence on how gender plays a role in presenting or disclosing health concerns in primary care. Specifically, our findings suggest that male patients may be reluctant to present certain RfEs to male GPs. Acknowledging these gender effects in primary care may help strengthen the partnership between GPs and patients.

We have also revised certain paragraphs in the introduction to clarify the topic and the relationship between gender and medical issues.

[Line 76, Introduction]:

'One interesting way to analyse this gender gap is to investigate reasons for encounter (RfE), particularly symptoms and complaints, as they reflect what prompts men and women to seek care and what they decide to disclose to their GPs. In other words, RfEs provide insight into how individuals evaluate and prioritize health concerns in their decision-making process to seek help'.

[Line 102, Introduction]:

'Previous studies investigating the effect of patients' and GPs' gender on RfE usually present results based on one or the other, but rarely together. As both seem to influence the care process, the challenge lies in distinguishing how they operate within a wide range of RfEs presented in primary care. Given that men consult less frequently and are often diagnosed at later stages of disease, it is important to disentangle whether GP gender may act as a barrier for men in disclosing certain health problems'.

Reviewer #2: The article provides a relevant analysis of gender differences in reasons for encounter in primary care, based on a large and representative dataset from France. The findings are valuable and the manuscript is well structured.

However, it is important for the authors to clarify that the results are not directly generalizable to the present context, since gender ideologies and the distribution of male and female physicians have changed in the last decade. In addition, the manuscript would benefit from a deeper consideration of the interaction between gender and age, for example discussing whether younger men and older men differ in consultation patterns, or explaining why this aspect was not explored.
Overall, the study is suitable for publication and represents a relevant contribution, but it would be strengthened by these additions to the discussion.

We'd like to thank the reviewer for this valuable comment. We agree with the fact that our study is likely not generalizable to the present context and that we lacked a more engaged discussion about age and gender interaction. We added these elements in the limitation section:

"Regarding our analyses, for simplicity reasons, our models were adjusted for patients' age, but this did not allow us to assess the interaction between patients' age and gender when presenting a given RfE. Indeed, it is possible that younger men or women have different attitudes towards GPs when presenting their health problems compared to older counterparts. We believe further studies should address this gap, especially considering how cohort effects regarding gender attitudes have changed in recent years."

About the generalizability to our current context:
"Finally, the analysed data were collected over 10 years prior to our study. Since then, many changes in society and medical demographics have occurred, including a potential increase in gender awareness among patients and GPs, as well as the continued feminization of the GP population (31), which makes our results less generalizable to the current context. At the same time, the number of GPs in France declined in recent years until 2021 (32). Although more recent data suggest a slight increase since then (31), the reduced density of GPs per capita (33) may lead people to consult a GP based on availability. For these reasons, the gender of GPs may have less impact on RfEs than before, as people have fewer options. Still, using data from the early 2010s, when this density was higher than it is nowadays (155 GPs and 146 GPs per 100,000 persons in 2012 and 2025, respectively (34)), can be more reliable for exploring gender effects in the presentation of RfE in primary care. In this regard, we argue that our findings, though not current, may be closer to true patient preferences, as access was easier back then."

Reviewer #3: Abstract:
1. Line 27- You should show the (abbreviation) for general practitioner (GP). Line 31—You use GP, but it is not defined earlier.
2. Line 34 – Capitalize "To."
We'd like to thank the reviewer for these remarks. We have corrected those sentences in the abstract.

Introduction
1. Recommend that authors provide background on the French healthcare system. For example, do patients have the flexibility to visit different GPs? What are insurance restrictions,

if any, on seeking providers? You provide some insight into the health system now in the strengths and limitations section, but I recommend providing some details earlier in the paper.

“In France, GPs act as the gatekeepers of the healthcare system, meaning that people are encouraged to visit a GP first, who will then refer them to specialized care if needed. People are also required to register with one GP (or other physician specialty), a measure intended to facilitate coordination and continuity throughout the care pathway. Unlike in other countries with gatekeeper systems, the French system adopts a liberal approach: people can choose to consult a different physician than the registered one, although they will be less reimbursed by the social security system (16). This implies that, to some extent, people can choose any GP for any given consultation. Even though many factors can play in this choice, starting from availability, we advance that, when possible, people may choose a GP for his or her gender for a specific problem.”

Methods

1. Clarify if the GP was present while the intern collected data on RfE and/or symptoms and complaints.

Thank you for this point. First sentence of the Data collection section states:

“Data were collected by 54 interns, observing their supervisors’ general practice.”

2. Were these first visits to the particular GP? Did the survey include a question specific to the number of times the patient has seen this particular GP, and if these were follow-up visits related to the chief health complaint? If so, adding these variables to your analysis could further explain gender preferences.

Thank you for these clarification questions. The survey did include a question regarding whether or not the patient was already known to this particular GP. This variable was included as a covariate in the multivariate models (“Established patient”). There was no question, however, about the number of times they had visited before, or the motives for previous consultations. We added a sentence in the limitation section.

‘Also, other variables that could have explained gender differences in presentation rates were not measured, such as the number of times patients had visited before a given GP, or the RfE for previous consultations.’

3. Please explain/state whether or not incentives were given to the GPs, interns, or patients for participating in the data collection?

Thank you for this point. Neither interns nor supervisors nor patients receive an incentive. We have added a sentence in the Data design section:

“No incentives were given to patients, interns, or GPs for participating in this study”.

Discussion

1. Recommend that authors address whether the gender of the intern made a difference in patient communicated complaint or RfE, in addition to the gender of the supervising GP.

We thank the reviewer for this remark. It would have been indeed very interesting; unfortunately, the gender of the intern was not included in the database. However, patients had the possibility to ask the intern not to be present, while still participating in the study, limiting the risk of not communicating complaints because of his or her presence. We have added this to the limitation section of the discussion.

“Similarly, the gender of the intern was not reported in the survey, so we could not assess whether it influenced the RfE. Nevertheless, patients could refuse the presence of the intern, which would limit the risk of bias in reporting RfE to their GPs.”

Conclusion
2. Suggest that the authors state that future studies, collecting data over several time points, could explore whether patients remained with their gender-preferred GP once the health complaint resolved.
Thank you for this valuable suggestion. We added a sentence in the conclusion:

“Future studies should consider adopting a longitudinal design to explore how GP-patient gender dyads evolve over time, particularly once a specific health complaint is resolved. This could shed light on whether the preferred GP gender remains consistent beyond the original RfE.”

==

IMPORTANT: PLEASE DO NOT REPLY TO THIS EMAIL

If you are unable to complete any points that are requested in this email, please explain why in the 'Enter Comments' tab of the online submission form prior to re-submitting your manuscript. This will let us quickly assess your response and send your manuscript to an Academic Editor as soon as possible.

==

PONE-D-25-38524R1
Reasons for encounter in primary care among French patients: gender differences in presentation rates more pronounced among the patients of male practitioners
Doctor Colinne Esther Patrice

Dear Dr. Patrice,

We've checked your submission and before we can proceed, we need you to address the following issues:

1. In the ethics statement in the Methods, you have specified that verbal consent was obtained. Please provide additional details regarding how this consent was documented and witnessed, and state whether this was approved by the IRB

Thank you for this point.
The IRB approval for this study explicitly permitted the use of oral informed consent, given the non-interventional and minimal-risk nature of the research in routine general practice. The IRB did not require written documentation or a witness signature. Oral consent was obtained by GPs and interns after informing patients about the study and its voluntary nature.
We have now revised the Ethics Statement in the methods section of the manuscript to clarify these points:

[Line 141-145] Further, the ECOGEN survey was approved by Ethics Committee Sud-Est IV (No.L11-149), which explicitly approved the oral form of consent obtained from patients given the observational and minimal-risk nature of the study. In accordance with the Ethics Committee's requirements, no written documentation or witness signature of the oral consent was requested or required.

2. Thanks for getting back to us about your data availability statement. You have mentioned that restrictions apply, and the data is available upon request. As part of our data policy, PLOS only allows for data to be accessed upon request if there are legal or ethical restrictions on sharing data publicly. Please refer to our webpage for more information (https://emea01.safelinks.protection.outlook.com/?url=http%3A%2F%2Fjournals.plos.org%2Fplosone%2Fs%2Fdata-availability%23loc-unacceptable-data-access-restrictions&data=05%7C02%7C%7C2d31030d36a3421fb2e708de28c3ef54%7C84df9e7fe9f640afb435aaaaaaaaaaaa%7C1%7C0%7C638993019173681392%7CUnknown%7CTWFpbGZsb3d8eyJFbXB0eU1hcGkiOnRydWUsIlYiOiIwLjAuMDAwMCIsIlAiOiJXaW4zMiIsIkFOIjoiTWFpbCIsIldUIjoyfQ%3D%3D%7C0%7C%7C%7C&sdata=Tg39%2FAGgn7I25qfRSZKctzWU33RDeaJUkovt1WdUcRw%3D&reserved=0).

In line with our goal of ensuring long-term data availability to all interested researchers, PLOS' Data Policy also states that authors cannot be the sole named individuals responsible for ensuring data access (https://emea01.safelinks.protection.outlook.com/?url=http%3A%2F%2Fjournals.plos.org%2Fplosone%2Fs%2Fdata-availability%23loc-acceptable-data-sharing-methods&data=05%7C02%7C%7C2d31030d36a3421fb2e708de28c3ef54%7C84df9e7fe9f640afb435aaaaaaaaaaaa%7C1%7C0%7C638993019173711022%7CUnknown%7CTWFpbGZsb3d8eyJFbXB0eU1hcGkiOnRydWUsIlYiOiIwLjAuMDAwMCIsIlAiOiJXaW4zMiIsIkFOIjoiTWFpbCIsIldUIjoyfQ%3D%3D%7C0%7C%7C%7C&sdata=TLh3TkR4vMp4t1PfgZPmP4pwzqKlGXn%2FDYoFUepphIo%3D&reserved=0).

Before we proceed with your manuscript, please address the following prompts:

a) If there are ethical or legal restrictions on sharing a de-identified data set, please explain them in detail (e.g., data contain potentially identifying or sensitive patient information) and who has imposed them (e.g., a Research Ethics Committee or Institutional Review Board, etc.). Please provide non-author contact information (phone/email/hyperlink) for a data access committee, ethics committee, or other institutional body to which data requests may be sent. If applicable, please also provide any necessary information that interested

researchers would need when requesting access to data to obtain the minimal dataset for your study.

Thank you for your comment. The data used in this study are subject to legal restrictions under French data protection regulations and cannot be publicly shared. We have now revised the Data Availability Statement to address these restrictions, to identify the authority responsible (CNIL), and to provide a non-author institutional contact for data access requests, in accordance with PLOS ONE requirements.
Please let us know if you require any additional clarification.

[Line 367] The data used in this study contain sensitive health information and are subject to legal restrictions under French data protection regulations. As required by the French Data Protection Authority (CNIL), these data cannot be publicly shared. Qualified researchers may request access to the minimal dataset from the Collège Universitaire de Médecine Générale of Lyon 1 University (dmg.cumg[at]univ-lyon1.fr). The institution guarantees persistent data storage and is responsible for maintaining availability for qualified researchers who meet the legal and ethical requirements.

b)Please also provide non-author contact information (phone/email/hyperlink) for a data access committee, ethics committee, or other institutional body to which data requests may be sent. If no institutional body is available to respond to requests for your minimal data, please consider if there are any institutional representatives who did not collaborate in the study, and are not listed as authors on the manuscript, who would be able to hold the data and respond to external requests for data access? If so, please provide their contact information (i.e., email address). Please also provide details on how you will ensure persistent or long-term data storage and availability.

Thank you for the clarification. We have now added the required non-author contact information for data access requests, as well as details on long-term data storage and availability, in the revised Data Availability Statement.
The institution responsible for the access to the Dataset is the Collège Universitaire de Médecine Générale of Lyon 1 University, they can be contacter at the address: dmg.cumg[at]univ-lyon1.fr

We've returned your manuscript to your account. Please resolve these issues and resubmit your manuscript within 21 days. If you need more time, please email the journal office at plosone@plos.org. We are happy to grant extensions of up to one month past this due date. If we do not hear from you within 21 days, we will withdraw your manuscript.

Please log on to PLOS Editorial Manager at https://emea01.safelinks.protection.outlook.com/?url=https%3A%2F%2Fwww.editorialmanager.com%2Fpone%2F&data=05%7C02%7C%7C2d31030d36a3421fb2e708de28c3ef54%7C84df9e7fe9f640afb435aaaaaaaaaaaa%7C1%7C0%7C638993019173731629%7CUnknown%7CTWFpbGZsb3d8eyJFbXB0eU1hcGkiOnRydWUsIlYiOiIwLjAuMDAwMCIsIlAiOiJXaW4zMiIsIkFOIjoiTWFpbCIsIldUIjoyfQ%3D%3D%7C0%7C%7C%7C&sdata=6DKlRiMSKPerj06xEXR0xk%2BIy%2BCoXxm%2B6gG79IejHvo%3D&reserved=0 to access your manuscript. You will find your manuscript in the 'Submissions Sent Back to Author' link under the New

Submissions menu. Be sure to remove your previous manuscript file if you are uploading a new file in response to these requests. After you've made the changes requested above, please be sure to view and approve the revised PDF after rebuilding the PDF to complete the resubmission process.

We are requesting these changes to comply with the PLOS ONE submission guidelines (https://emea01.safelinks.protection.outlook.com/?url=https%3A%2F%2Fjournals.plos.org%2Fplosone%2Fs%2Fsubmission-guidelines&data=05%7C02%7C%7C2d31030d36a3421fb2e708de28c3ef54%7C84df9e7fe9f640afb435aaaaaaaaaaaa%7C1%7C0%7C638993019173752210%7CUnknown%7CTWFpbGZsb3d8eyJFbXB0eU1hcGkiOnRydWUsIlYiOiIwLjAuMDAwMCIsIlAiOiJXaW4zMiIsIkFOIjoiTWFpbCIsIldUIjoyfQ%3D%3D%7C0%7C%7C%7C&sdata=ph%2BBzwJsCb2etqTNpDsNvv1ArI5vIoNkOAX3FYX%2Bay4%3D&reserved=0). Please note that we won't send your manuscript for review until you have resolved the above requests.

Thank you for submitting your work to PLOS ONE and supporting our mission of Open Science.

Kind regards,

Jeunarine Repe Flores
PLOS ONE

==

PONE-D-25-38524R1
Reasons for encounter in primary care among French patients: gender differences in presentation rates more pronounced among the patients of male practitioners
Doctor Colinne Esther Patrice

Dear Dr. Patrice,

We've checked your submission and before we can proceed, we need you to address the following issues:

Thanks for getting back to us about your data availability statement. You have mentioned that the data is available by request at the Université Claude Bernard
Lyon 1 (UCBL) because they are stored on the UCBL servers.

Before we proceed, we'll require some additional clarification to ensure your submission adheres to the PLOS ONE Data Availability policy (https://emea01.safelinks.protection.outlook.com/?url=https%3A%2F%2Fjournals.plos.org%2Fplosone%2Fs%2Fdata-availability&data=05%7C02%7C%7C3be0b24b8f60493ef03008de3ba84cfa%7C84df9e7fe9f

640afb435aaaaaaaaaaaa%7C1%7C0%7C639013791205459567%7CUnknown%7CTWFpbGZsb3d8eyJFbXB0eU1hcGkiOnRydWUsIlYiOiIwLjAuMDAwMCIsIlAiOiJXaW4zMiIsIkFOIjoiTWFpbCIsIldUIjoyfQ%3D%3D%7C0%7C%7C%7C&sdata=z%2FlX0TbnCNs9fZ2RGBNqYyVisXngtBNKK6PsL2InW08%3D&reserved=0).

Please note that the data we ask authors to share is not necessarily the full and/or raw data set collected in the course of their research, but rather the "minimal data set", which we define as consisting of the data set used to reach the conclusions drawn in the manuscript with related metadata and methods, and any additional data required to replicate the reported study findings in their entirety.

Depending on the nature of your study, this can include any or all of the following:

- The values behind the means, standard deviations and other measures reported;

- The values used to build graphs;

- The points extracted from images for analysis.

For studies involving third-party data (i.e., data not owned or collected by the authors), we encourage authors to share any data specific to their analyses that they can legally distribute. PLOS recognizes, however, that authors may be using third-party data they do not have the rights to share. When third-party data cannot be publicly shared, authors must provide all information necessary for interested researchers to apply to gain access to the data in the text of the Data Availability statement. This statement will be published online alongside your article should it be accepted for publication.

For any third-party data that the authors cannot legally distribute, please provide the following information in an updated and complete Data Availability statement:

a) Please kindly explain in detail how others can access the minimal datasets you used in this study from the third-party websites you accessed. Please provide any relevant information necessary to gain immediate access to the specific minimal datasets used in your work (e.g. contact information, specific URLs, dataset titles, etc.).

The minimal dataset used for this study can be accessed specifically by request to the RESearch on HealthcAre Performance (RESHAPE) research unit of the University Lyon 1, tied to the College of Medecine General, at the mail adress : reshape@univ-lyon1.fr.

b) Please clarify in detail whether interested researchers can replicate your study findings in their entirety by directly obtaining the data from the third party sources in your Data Availability statement and following the information outlined in your Methods section.

Interested researcher can replicate the study findings in their entirety by obtaining the minimum dataset from the RESHAPE unit and follow the information outlined in the Method section of our article.

c) Please also confirm whether the authors had any special access privileges that others would not have when attempting to access the minimal data you used from these third party sources.

As we were working with Pr Laurent Letrillart, who is part of the RESHAPE unit and the original ECOGEN survey, we were given access to the full ECOGEN database, including data we did not use for our study. This might not be the case for others who require access to the database, as this is proprietary data. Also the complete ECOGEN database is pseudo-anonymised and so is considered personal data by the CNIL, therefore submitted to french regulation General Data Protection Regulation (GRPD). The GRPD does not allow transferrance of data oustide the Europe Union, unless the country of reception can garanty the same level of data protection.
However the minimal anonymous dataset necessary to replicate the results of our study is not subjected to those restriction.

Alternatively, if there are no restrictions, please upload the complete minimal anonymized data set necessary to replicate your study findings to a stable, public repository and provide us with the relevant URLs, DOIs, or accession numbers.

For a list of recommended repositories, please see:
https://emea01.safelinks.protection.outlook.com/?url=https%3A%2F%2Fjournals.plos.org%2Fplosone%2Fs%2Frecommended-repositories&data=05%7C02%7C%7C3be0b24b8f60493ef03008de3ba84cfa%7C84df9e7fe9f640afb435aaaaaaaaaaaa%7C1%7C0%7C639013791205486210%7CUnknown%7CTWFpbGZsb3d8eyJFbXB0eU1hcGkiOnRydWUsIlYiOiIwLjAuMDAwMCIsIlAiOiJXaW4zMiIsIkFOIjoiTWFpbCIsIldUIjoyfQ%3D%3D%7C0%7C%7C%7C&sdata=RC7md%2FPgzvyQK3ViGLS%2FC0jd%2FW%2FbIVdETDDV0Ns63xQ%3D&reserved=0

You also have the option of uploading the data as Supporting Information files, but we would recommend depositing data directly to a data repository if possible.

We will update your Data Availability statement on your behalf to reflect the information you provide.

We've returned your manuscript to your account. Please resolve these issues and resubmit your manuscript within 21 days. If you need more time, please email the journal office at plosone@plos.org. We are happy to grant extensions of up to one month past this due date. If we do not hear from you within 21 days, we will withdraw your manuscript.

Please log on to PLOS Editorial Manager at https://emea01.safelinks.protection.outlook.com/?url=https%3A%2F%2Fwww.editorialmanager.com%2Fpone%2F&data=05%7C02%7C%7C3be0b24b8f60493ef03008de3ba84cfa%7C84df9e7fe9f640afb435aaaaaaaaaaaa%7C1%7C0%7C639013791205501215%7CUnknown%7CTWFpbGZsb3d8eyJFbXB0eU1hcGkiOnRydWUsIlYiOiIwLjAuMDAwMCIsIlAiOiJXaW4zMiIsIkFOIjoiTWFpbCIsIldUIjoyfQ%3D%3D%7C0%7C%7C%7C&sdata=FN%2B2%2BNP6%2B6CnqPY9%2BWHq0of6WDfOhftdqjhB66bAcSU%3D&reserved=0 to access your manuscript. You will find your manuscript in the 'Submissions Sent Back to Author' link under the New Submissions menu. Be sure to remove your previous manuscript file if you are uploading a new file in response to these requests. After you've made the changes requested above, please be sure to view and approve the revised PDF after rebuilding the PDF to complete the resubmission process.

We are requesting these changes to comply with the PLOS ONE submission guidelines (https://emea01.safelinks.protection.outlook.com/?url=https%3A%2F%2Fjournals.plos.org%2Fplosone%2Fs%2Fsubmission-guidelines&data=05%7C02%7C%7C3be0b24b8f60493ef03008de3ba84cfa%7C84df9e7fe9f640afb435aaaaaaaaaaaa%7C1%7C0%7C639013791205519018%7CUnknown%7CTWFpbGZsb3d8eyJFbXB0eU1hcGkiOnRydWUsIlYiOiIwLjAuMDAwMCIsIlAiOiJXaW4zMiIsIkFOIjoiTWFpbCIsIldUIjoyfQ%3D%3D%7C0%7C%7C%7C&sdata=1g5athA%2FP6EYjSyfK2FI77g9fsfGCWHBsvQt9xTY2Uo%3D&reserved=0). Please note that we won't send your manuscript for review until you have resolved the above requests.

Thank you for submitting your work to PLOS ONE and supporting our mission of Open Science.

Kind regards,

Khem Descatamiento
PLOS One
